# From PPG to Blood Pressure at the Edge: Quantization-Aware Architecture Selection and On-MCU Validation

**DOI:** 10.3390/s26092674

**Published:** 2026-04-25

**Authors:** Elisabetta Leogrande, Emanuele De Luca, Francesco Dell’Olio

**Affiliations:** Micro Nano Sensor Group, Polytechnic University of Bari, Via Edoardo Orabona 4, 70126 Bari, Italy; e.leogrande1@phd.poliba.it (E.L.); e.deluca3@phd.poliba.it (E.D.L.)

**Keywords:** cuffless blood pressure estimation, photoplethysmography, quantization-aware design, TinyML, embedded neural networks, STM32N6

## Abstract

Blood pressure is a central marker of cardiovascular risk, but continuous monitoring remains difficult because cuff-based measurements are intermittent and uncomfortable. Photoplethysmography (PPG) is already ubiquitous in wearables and can, in principle, enable cuffless blood pressure estimation from a single optical signal. However, many deep learning approaches that perform well in floating-point are impractical for microcontroller-class devices, where memory budgets, latency, and integer-only arithmetic constrain what can be deployed. A key open question is which neural architectures retain accuracy after full-integer quantization, rather than only under desktop inference. Here, we show an end-to-end, microcontroller-oriented evaluation framework that benchmarks multiple 1D convolutional models for cuffless systolic and diastolic pressure estimation from single-channel PPG, jointly optimizing estimation error, model footprint, and quantization robustness. We find that floating-point accuracy alone is a poor predictor of deployability: some lightweight CNNs exhibit substantial performance drift after INT8 conversion, whereas a compact residual 1D CNN preserves its predictions with near-identical error statistics after integer quantization. We then deploy the selected integer-only model on an STM32N6 microcontroller using an industrial toolchain and confirm that on-device inference maintains low bias and limited error dispersion while meeting real-time constraints for continuous operation. These results highlight architecture-dependent quantization stability as a critical design dimension for sensor-edge intelligence and support the feasibility of fully on-device cuffless blood pressure monitoring without multimodal sensing or cloud processing.

## 1. Introduction

Photoplethysmography (PPG) is a non-invasive optical technique that measures blood volume variations in microvascular tissue and is widely used for monitoring physiological parameters [1]. Owing to its simplicity, low cost, and ease of integration into wearable devices, PPG has become a standard tool for tracking heart rate, oxygen saturation, and, more recently, blood pressure (BP) [2].

Compared to alternative blood-pressure-monitoring approaches, PPG offers several distinct advantages: it is entirely non-invasive and painless, requires only a single low-cost optical sensor, and can be readily integrated into wearable form factors such as smartwatches, wristbands, and finger-worn rings, enabling continuous and unobtrusive monitoring without the discomfort of cuff inflation. Unlike multimodal approaches combining ECG and PPG to derive pulse transit time (PTT) or pulse arrival time (PAT), single-channel PPG methods eliminate the need for additional electrode placement, thereby reducing hardware complexity and improving user compliance [3,4,5]. However, PPG-based blood pressure estimation also presents inherent limitations. The PPG signal is sensitive to motion artifacts, changes in ambient light conditions, skin pigmentation, and variations in sensor-skin contact pressure, which can significantly alter waveform morphology and degrade estimation accuracy [6]. Furthermore, the relationship between PPG signal features and arterial blood pressure is indirect and subject-dependent, as it is mediated by vascular compliance, autonomic regulation, and other hemodynamic factors. Consequently, single-channel PPG-based methods generally exhibit higher estimation uncertainty compared to cuff-based or multimodal approaches, representing an inherent trade-off between measurement simplicity and estimation precision.

Continuous BP monitoring is essential for cardiovascular disease management, yet traditional cuff-based methods remain limited in comfort and continuity [7]. To overcome these constraints, recent research has focused on cuffless BP estimation using physiological signals such as PPG, often in combination with Electrocardiography (ECG) to derive pulse transit or arrival times [3,4,5]. However, multimodal approaches increase hardware and computational complexity, motivating the development of single-signal PPG methods based on morphological analysis [8,9,10], or deep neural networks that directly map raw waveforms to BP values [11,12,13,14].

Recent comprehensive reviews have highlighted that PPG-based cuffless BP estimation has rapidly evolved from feature-based and cloud-centric machine learning approaches toward deep learning solutions explicitly designed for edge and embedded deployment, driven by the need for real-time processing, reduced latency, and enhanced data privacy [15]. Deep learning frameworks such as PP-Net have demonstrated the feasibility of jointly estimating systolic and diastolic blood pressure directly from raw PPG signals [12]. Artificial intelligence (AI) has also shown promise for real-time physiological monitoring through Tiny Machine Learning (TinyML) models on microcontrollers [16]. However, many proposed solutions rely on complex neural architectures or multimodal datasets, limiting their applicability in low-power embedded devices [17]. This tension between algorithmic accuracy and deployability on constrained hardware has become a central challenge in the translation of cuffless BP estimation from research prototypes to real-world systems.

In this context, edge computing has emerged as a key paradigm for biomedical signal processing, enabling on-device inference that reduces dependence on cloud infrastructures while addressing concerns related to data privacy, communication latency, and energy consumption [15]. For wearable and point-of-care applications, these aspects are particularly critical, as continuous wireless data transmission may significantly impact battery lifetime and raise regulatory and privacy concerns. Consequently, there is growing interest in developing end-to-end edge AI pipelines capable of acquiring, processing, and interpreting physiological signals directly on resource-constrained hardware platforms. From a system-design perspective, this shift places strict constraints not only on model accuracy but also on memory footprint, inference latency, and energy efficiency.

In particular, recent studies have emphasized that although deep learning architectures such as convolutional, recurrent, and transformer-based networks can achieve high BP estimation accuracy, their direct deployment on resource-constrained microcontrollers remains challenging due to memory footprint, inference latency, and power consumption constraints [18]. Recent studies demonstrate that compression techniques such as quantization, pruning, and model optimization can substantially reduce model size while maintaining clinically acceptable accuracy, enabling practical AI deployment at the edge [19,20]. However, despite these advances, reported performance gains are often evaluated in isolation from the target hardware, overlooking the tight coupling between model design and the characteristics of the deployment platform.

Quantization-aware and post-training quantization strategies have been shown to reduce model size by up to an order of magnitude, enabling inference on edge devices while preserving BP estimation performance within clinical tolerance limits. Nevertheless, most existing works reporting aggressive model compression evaluate performance on desktop-class processors, GPUs, or general-purpose edge devices, without providing experimental validation on microcontroller-class platforms. As highlighted by recent benchmarking efforts in TinyML, performance metrics such as inference latency and memory footprint are highly hardware-dependent and cannot be reliably extrapolated without on-device measurements [18].

Advances in PPG-based BP estimation include IoT-oriented implementations using hybrid CNN–LSTM architectures achieving sub-2 mmHg errors [17] and CNN–SVR models leveraging multiwavelength PPG [21]. Further studies have shown that physiological and acquisition-related factors, such as PPG sensor contact pressure, can significantly alter waveform morphology and impact cuffless BP estimation performance [6]. Additional PPG-based deep learning approaches have been explored in wearable and edge-oriented scenarios, demonstrating the applicability of convolutional neural networks for real-time physiological signal analysis, although often targeting tasks other than blood pressure estimation and without validation on microcontroller-class hardware.

More broadly, recent surveys focusing on cuffless BP monitoring and edge-AI-enabled healthcare systems have emphasized the lack of end-to-end experimental validation on embedded microcontrollers, identifying model generalization, deployment constraints and hardware-aware optimization as key open challenges for real-world adoption [15].

Nevertheless, most existing works depend on high-complexity networks or desktop-class hardware, leaving a gap in demonstrating clinically compliant BP estimation on microcontrollers operating under strict memory and latency constraints. This study contributes to addressing this gap by investigating whether a compact CNN, trained on a public dataset and deployed on a real MCU, can achieve estimation performance aligned with commonly adopted reference benchmarks derived from AAMI validation criteria, without relying on multimodal inputs or cloud resources.

The contribution of this work extends beyond a comparative benchmarking of existing CNN variants. Specifically, the novelty of the proposed approach lies in three key aspects. First, we introduce a structured, end-to-end methodology that jointly evaluates estimation accuracy, quantization robustness, and on-device feasibility as interconnected design dimensions, rather than treating them independently as is common in the literature. Second, we demonstrate that floating-point accuracy is a poor predictor of post-quantization deployability, establishing architecture-dependent quantization stability as a first-class design criterion for embedded physiological monitoring. Third, we provide one of the few experimental validations of cuffless PPG-based BP estimation on a microcontroller-class platform (STM32N6), closing the loop from algorithm design to real-world embedded deployment. These aspects collectively distinguish the present work from standard model-comparison studies and position it as a contribution to the emerging field of hardware-aware design for edge health monitoring.

The objective of this paper is to investigate the feasibility of cuffless blood pressure estimation using a single photoplethysmography signal within a fully embedded, microcontroller-oriented framework. In particular, this study aims to identify neural network architectures that simultaneously achieve clinically acceptable accuracy, robustness to full integer quantization, and compatibility with the memory and latency constraints of microcontroller-class hardware. To this end, multiple convolutional neural network models are systematically compared, and the most suitable architecture is selected, quantized, and validated through both off-device and on-device experiments. The remainder of this paper is organized as follows: Section 2 describes the dataset and the preprocessing pipeline adopted for photoplethysmography signal conditioning, together with the neural network architectures considered in this study and the evaluation methodology used for performance assessment and model selection. Section 3 presents the experimental results obtained during off-device evaluation, including the comparison of floating-point and quantized models, as well as the analysis of accuracy, robustness to quantization, and memory footprint. Furthermore, it reports the on-device deployment and validation of the selected model on an STM32N6 microcontroller, discussing inference performance, memory occupation, and practical feasibility for embedded execution. Finally, Section 4 provides a discussion of the results in the context of related embedded blood pressure estimation approaches and concludes this paper by outlining the main contributions and future research directions.

## 2. Materials and Methods

Experiments were conducted using the publicly available UCI “Cuff-Less Blood Pressure Estimation” dataset [22], which provides simultaneous recordings of infrared photoplethysmography (PPG) and arterial blood pressure (ABP) signals. The four partitions (Part1–Part4) were merged into a single dataset. Following the preprocessing criteria adopted in previous work [23,24], only recordings with a valid channel and a minimum duration of eight minutes were retained, while signals exhibiting abnormal ABP peaks above 200mmHg were excluded. As a result of this selection, only 500 valid signals remained, and to ensure uniformity across the experiments, the analysis was therefore limited to these records [23].

**Figure 1:**
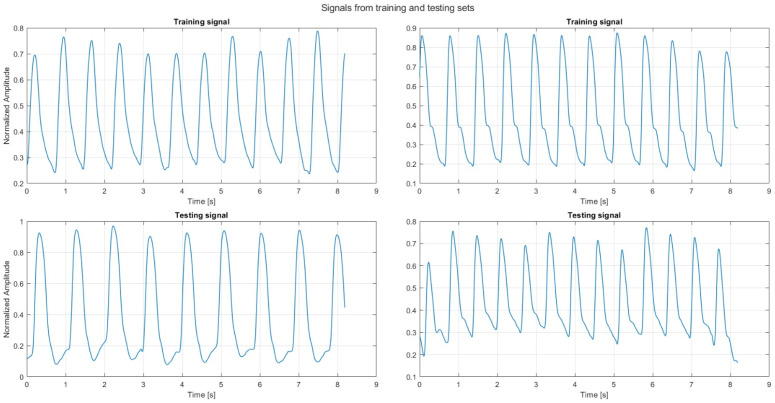
Examples of normalized infrared PPG segments extracted from the UCI dataset, showing representative signals from both training (**top**) and testing (**bottom**) partitions.

The PPG channel was detrended to remove baseline wander and normalized using standard min–max scaling to the range [0, 1], as illustrated in Figure 1. The ABP signal was segmented into 8.192s windows (1024 samples at 125Hz) with 75% overlap [23], from which SBP and DBP were extracted as the maximum and minimum values. Each training instance therefore consisted of a normalized PPG segment paired with systolic and diastolic pressures in mmHg.

**Figure 2:**
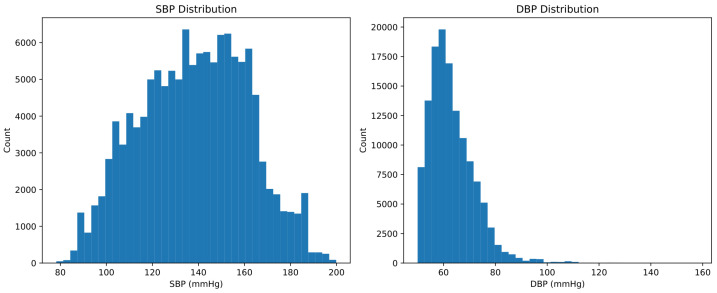
Distribution of systolic and diastolic blood pressure values in the full dataset.

This segmentation procedure generated a total of 129,276 windowed instances, which were randomly partitioned into 90,493 training windows (70%) and 38,783 test windows (30%). To mitigate potential leakage due to overlapping segmentation, identical segments across splits were removed prior to final evaluation. Across the entire dataset, SBP values ranged from 78.25 to 199.87mmHg (median 139.01mmHg), while DBP values ranged from 50.00 to 158.02mmHg (median 61.49mmHg). The corresponding distributions are illustrated in Figure 2.

It should be noted that the adopted partitioning strategy assigns windows to training and test sets randomly, without enforcing subject-level disjointness. As a result, windows originating from the same subject, or even from the same recording session, may appear in both sets. Combined with the 75% overlap used during segmentation, this may introduce a degree of data leakage that could lead to optimistic error estimates compared to a strict subject-wise or leave-one-subject-out evaluation protocol. This partitioning choice was adopted for consistency with the preprocessing pipelines of prior studies on the same dataset [23,24], and to enable a controlled comparison among architectures under identical conditions. The implications of this choice for clinical generalizability are discussed in Section 4.

Figure 2 characterizes the blood pressure distributions underlying all subsequent analyses. The SBP distribution is approximately bell-shaped but spans a broad range (78–200 mmHg), providing the models with a relatively uniform representation of systolic values during training. In contrast, the DBP distribution is markedly right-skewed, with a heavy concentration in the 55–70 mmHg range and a long tail extending above 100 mmHg. This asymmetry has direct implications for model training, as the network is exposed to a substantially imbalanced distribution for diastolic pressure, and for the interpretation of error metrics, since prediction errors may differ across well-represented and underrepresented blood pressure ranges.

All preprocessing operations, including record selection, detrending, normalization, window segmentation, and dataset splitting, were implemented in MATLAB R2024b (MathWorks, Natick, MA, USA), while training and validation of the neural networks (NNs) were carried out in TensorFlow 2.16 (Google LLC, Mountain View, CA, USA) on Google Colab.

### 2.1. Neural Network Architectures Considered

To systematically evaluate the impact of architectural design choices on cuffless blood pressure estimation, several neural network models were implemented and benchmarked using the same dataset, preprocessing pipeline, and evaluation protocol. The tested architectures differ in depth, connectivity, and convolutional strategy, while preserving a comparable input representation and training setup in order to enable a fair and controlled comparison.

The first model, referred to as Baseline 1D CNN, is a plain one-dimensional convolutional neural network composed of stacked convolutional layers followed by fully connected regression layers. This architecture does not employ residual connections or specialized convolutional operators and serves as a reference point to assess the limitations of standard CNN designs when applied to PPG-based blood pressure estimation. The network consists of a sequence of Conv1D layers with progressively increasing channel width, each followed by nonlinear activation and temporal downsampling through pooling operations, and terminates in a dense regression head producing systolic and diastolic blood pressure estimates.

A second architecture, denoted as *Residual 1D CNN*, extends the baseline design by introducing residual connections between convolutional blocks. These skip connections facilitate gradient propagation during training and improve representational stability, allowing the network to achieve higher accuracy without a proportional increase in depth or parameter count. The model is organized into residual stages in which each block comprises two convolutional layers with identity shortcut connections. Channel dimensionality increases across stages, enabling hierarchical temporal feature extraction while maintaining stable optimization.

To improve suitability for embedded deployment, a compressed variant named *Residual 1D CNN–Slim* was also evaluated. This model preserves the residual structure while reducing the number of channels and overall parameter count, aiming to balance estimation performance with memory footprint and computational complexity. Compared to the larger residual architecture, the Slim variant reduces the number of filters per stage and adopts a compact regression head. A global average pooling layer is employed before the final dense layer to limit the number of trainable parameters and improve architectural compactness.

The fourth tested architecture is *MobileNet-1D*, which replaces standard convolutions with depthwise separable convolutions. This design significantly reduces the number of parameters and arithmetic operations, making it attractive for low-power platforms, while potentially impacting robustness in regression tasks. In this configuration, each convolutional stage is decomposed into a depthwise temporal convolution followed by a pointwise convolution, separating temporal filtering from channel mixing and substantially reducing computational complexity.

Finally, a compact Micro Temporal Convolutional Network (TCN) was included to explore sequence-oriented convolutional designs based on dilated convolutions. This architecture emphasizes temporal receptive field expansion with a minimal number of parameters. Dilated one-dimensional convolutions are arranged within residual structures, allowing the effective receptive field to grow without a proportional increase in parameter count, thereby enabling efficient modeling of long-range temporal dependencies in PPG signals.

Across all architectures, the ReLU activation function was used after each convolutional layer. Temporal downsampling was performed via MaxPooling1D operations in the Baseline and Residual models, while the MobileNet-1D and Micro TCN relied on strided convolutions for dimensionality reduction. All models share an input shape of (1024,1), corresponding to a single-channel PPG window of 8.192s at 125Hz, and produce a two-element output vector representing the estimated SBP and DBP values. The Baseline 1D CNN employs four convolutional layers with progressively increasing filter counts, followed by a flatten operation and two dense layers. The Residual 1D CNN is organized into four residual stages, each consisting of two convolutional layers with identity shortcut connections and increasing channel dimensionality. The Residual 1D CNN–Slim adopts the same residual structure with reduced filter counts per stage and replaces the flatten operation with global average pooling (GAP) to minimize the number of parameters in the regression head. The MobileNet-1D decomposes each convolutional stage into a depthwise convolution (operating along the temporal axis) followed by a 1×1 pointwise convolution for channel mixing. The Micro TCN employs dilated causal convolutions with exponentially increasing dilation factors arranged within residual blocks, enabling broad temporal receptive fields with minimal parameter overhead.

Table 1 summarizes the main architectural and training characteristics of the evaluated neural network models. The Baseline 1D CNN is intentionally configured using a simple and generic training setup and serves as a reference model, whereas the remaining architectures adopt a unified, hardware-aware training strategy designed to better reflect embedded deployment constraints.

The total number of trainable parameters for each model is reported in Table 2.

All models were trained using the same input representation and dataset partitioning protocol. The training subset was further divided into training and validation sets using an 80/20 ratio with shuffling enabled and a fixed random state (random_state=42). A batch size of 128 was adopted across all experiments.

The Baseline 1D CNN was trained using the Adam optimizer (learning rate $=1\times 10^{-3}$) and mean squared error (MSE) loss for 400 fixed epochs. The remaining architectures were trained using the AdamW optimizer (learning rate $=8\times 10^{-4}$, weight decay $=1\times 10^{-4}$) and Huber loss. The Huber loss, also known as smooth $L_{1}$ loss, is defined as follows:

(1) $$L_{\delta }\left(e\right)=\left\{\begin{array}{cc} \frac{1}{2}\;e^{2}, & \mathrm{if}\;|e|\leq \delta \\ \delta \left(\left|e\right|-\frac{1}{2}\;\delta \right), & \mathrm{otherwise} \end{array}\right.$$

where $e=P_{i}^{\mathrm{ref}}-P_{i}^{\mathrm{est}}$ is the estimation error and δ is the transition threshold (set to 1.0 in this work). The Huber loss combines the stability of $L_{2}$ loss for small errors with the robustness of $L_{1}$ loss for large errors, reducing the influence of outlier samples during training.

Early stopping with restoration of the best validation weights was applied, with patience ranging from 30 to 35 epochs depending on the architecture. A ReduceLROnPlateau learning rate scheduler was used (factor =0.5, patience =10–12 epochs, minimum learning rate $=1\times 10^{-6}$). Each architecture was trained once under a fixed random seed (42), ensuring deterministic data shuffling and reproducibility of the reported results.

### 2.2. Methodology

To enable a fair and systematic comparison among the considered neural network architectures, a structured evaluation and selection methodology was adopted. All models were trained and tested using the same dataset, preprocessing pipeline, and data split in order to isolate the impact of architectural design choices on cuffless blood pressure estimation performance.

In a first stage, all five networks were evaluated in floating-point precision to assess their baseline estimation accuracy under identical training and testing conditions. Performance was quantified using clinically relevant error metrics for systolic and diastolic blood pressure, together with model size information, allowing for an initial screening based on accuracy and suitability for embedded deployment. Based on this analysis, two architectures were selected for further investigation, as they provided the most favorable trade-off between estimation performance and architectural complexity.

In a second stage, the selected models were subjected to post-training quantization in order to evaluate their robustness to reduced numerical precision. The consistency between floating-point and quantized inference was analyzed by comparing prediction errors and by assessing the correlation between the corresponding outputs. This step was designed to identify architectures capable of preserving estimation accuracy after quantization, a critical requirement for deployment on resource-constrained hardware.

It should be noted that each architecture was trained once using a fixed random seed, rather than across multiple independent runs. This choice was motivated by the need for strict reproducibility and by consistency with common practices in the TinyML and embedded-deployment literature, where the primary focus is on deployment feasibility rather than statistical benchmarking. Moreover, the use of early stopping with restoration of the best validation weights mitigates sensitivity to random initialization by selecting the model checkpoint that minimizes validation loss, regardless of the specific trajectory taken during training. Nonetheless, multi-seed evaluation would further strengthen the statistical robustness of the comparison and is recommended for future benchmarking studies.

Finally, the model exhibiting the highest robustness to quantization was deployed and tested on a real microcontroller platform using vendor-provided deployment tools. This last step enabled the validation of the complete end-to-end pipeline, from offline training to on-device inference, under realistic memory and execution constraints.

It should also be noted that the two architectures advanced to the quantization stage were selected on the basis of a joint accuracy-complexity criterion rather than purely on floating-point test accuracy. The subsequent analyses—post-training quantization, FP32 vs. INT8 consistency, and on-MCU deployment—constitute within-model comparisons aimed at assessing quantization-induced drift and deployment-induced numerical degradation, rather than establishing absolute estimation performance. Nonetheless, since the same held-out test set is used both for architecture selection and for the subsequent quantization and deployment evaluation, a mild form of model-selection bias cannot be excluded, and a fully independent test set would be required for formal clinical performance benchmarking.

**Figure 3:**
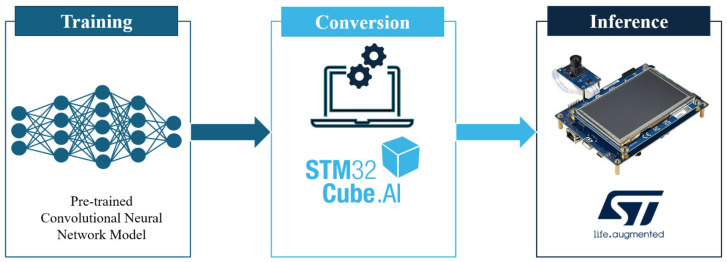
Embedded deployment workflow.

Figure 3 illustrates the end-to-end embedded deployment workflow adopted in this work, from offline network training to model conversion and on-device inference on the target microcontroller. The workflow comprises three stages. In the first stage, the CNN is trained in TensorFlow on a desktop or cloud environment using the preprocessed PPG dataset. In the second stage, the trained model is exported to TensorFlow Lite format and subjected to full-integer (INT8) post-training quantization; the resulting quantized model is then processed by the STM32Cube.AI toolchain, which generates optimized C code tailored for execution on the target STM32N6 microcontroller. In the third stage, the generated firmware is deployed on the MCU, where on-device inference is performed: the microcontroller receives PPG input windows, executes the quantized CNN using integer-only arithmetic, and produces SBP and DBP estimates in real time. This pipeline ensures that the complete chain from algorithm design to embedded execution is validated under realistic memory and computational constraints.

### 2.3. Evaluation Metrics and Performance Criteria

The performance of the proposed neural network architectures was evaluated using error metrics commonly adopted in cuffless blood pressure estimation and in accordance with the AAMI/ESH/ISO validation requirements [25]. Systolic blood pressure (SBP) and diastolic blood pressure (DBP) were analyzed separately.

For each test sample, the signed estimation error was defined as follows:

(2) $$e_{i}=P_{i}^{\mathrm{ref}}-P_{i}^{\mathrm{est}},$$

where $P_{i}^{\mathrm{ref}}$ and $P_{i}^{\mathrm{est}}$ denote the reference and estimated blood pressure values, respectively.

The mean error (ME) was computed as the average of the signed errors over the test set, providing a measure of systematic bias:

(3) $$\mathrm{ME}=\frac{1}{N}\sum_{i=1}^{N}e_{i}$$

The standard deviation (SD) of the signed error distribution was also calculated to quantify the variability in the estimation around the mean error:

(4) $$\mathrm{SD}=\sqrt{\frac{1}{N-1}\sum_{i=1}^{N}{\left(e_{i}-\mathrm{ME}\right)}^{2}}$$

As commonly adopted reference thresholds derived from the AAMI/ESH/ISO standard, values of |ME|≤5mmHg and SD<8mmHg are often used for retrospective comparison in cuffless BP estimation studies. It is important to note that the full AAMI/ESH/ISO universal standard [25] defines a validation protocol based on subject-level, prospective measurements with specific requirements on population diversity, reference device specifications, and minimum sample sizes, which are fundamentally different from the retrospective, window-level evaluation conducted in this study. Therefore, these criteria were used as commonly adopted reference benchmarks for comparability with the existing literature, rather than as evidence of formal clinical validation or AAMI/ESH/ISO compliance. This distinction should be kept in mind when interpreting the reported results.

In addition to ME and SD, the mean absolute error (MAE) was reported as a complementary metric to provide an intuitive measure of the average magnitude of the estimation error, irrespective of its sign:

(5) $$\mathrm{MAE}=\frac{1}{N}\sum_{i=1}^{N}\left|e_{i}\right|$$

While MAE is not part of the AAMI compliance criteria, it is widely used in the literature to facilitate comparison among different learning-based approaches and to improve result interpretability.

Model complexity was also taken into account during the evaluation process. In particular, the complexity of each network was quantified in terms of both the total number of trainable parameters and the model size, expressed as the storage required for the trained weights. This information was used together with the error metrics to assess the suitability of each architecture for deployment on resource-constrained embedded platforms.

These metrics collectively defined the performance criteria used in the subsequent selection and quantization stages, enabling a structured comparison between accuracy, robustness, and implementation cost.

## 3. Results

### 3.1. Floating-Point Network Comparison

The floating-point performance of the five considered neural network architectures was first evaluated in order to assess their baseline blood pressure estimation accuracy under identical experimental conditions. Table 2 summarizes the estimation results in terms of mean error (ME), standard deviation (SD) of the signed error, and mean absolute error (MAE) for both systolic and diastolic blood pressure. All metrics were computed on the independent test set defined in Section 2.

The Baseline 1D CNN achieves performance within commonly adopted reference thresholds. The residual architectures exhibit reduced error dispersion compared to non-residual models, with the large Residual 1D CNN achieving the highest estimation accuracy at the cost of increased model complexity. The Residual 1D CNN–Slim preserves most of the accuracy benefits of the larger model while significantly reducing architectural complexity, suggesting a more favorable trade-off for embedded deployment.

The five-model comparison implicitly constitutes an ablation-like analysis across several key design dimensions. Comparing the Baseline 1D CNN with the Residual 1D CNN isolates the contribution of residual connections: the introduction of skip connections reduces SBP SD from 7.38 to 3.35mmHg and DBP SD from 4.68 to 2.24mmHg, demonstrating a substantial improvement in estimation stability attributable to improved gradient propagation and representational stability. Comparing the Residual 1D CNN with the Residual 1D CNN–Slim isolates the effect of channel reduction (slimming): the Slim variant achieves a 4× reduction in parameter count (140,034 vs. 554,498) with only a moderate increase in SD (SBP: 4.57 vs. 3.35mmHg), confirming that aggressive architectural compression preserves most of the accuracy benefits. The MobileNet-1D architecture, which replaces standard convolutions with depthwise separable convolutions, yields a very small model (10,738 parameters, 37KB) but exhibits the highest SBP dispersion (SD =7.94mmHg), suggesting that depthwise separation may compromise representational capacity for regression tasks. Finally, the Micro TCN, based on dilated temporal convolutions, achieves intermediate performance with a compact footprint (41,698 parameters), demonstrating that expanded temporal receptive fields can partially compensate for limited model depth.

**Figure 4:**
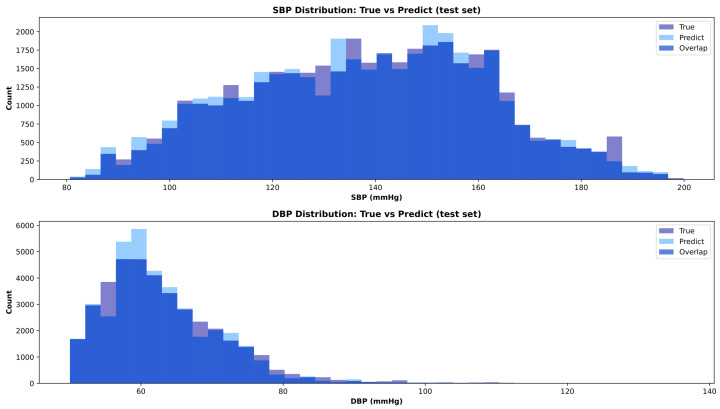
Distribution of true and predicted systolic and diastolic blood pressure values obtained with the Residual 1D CNN–Slim model on the independent test set.

**Figure 5:**
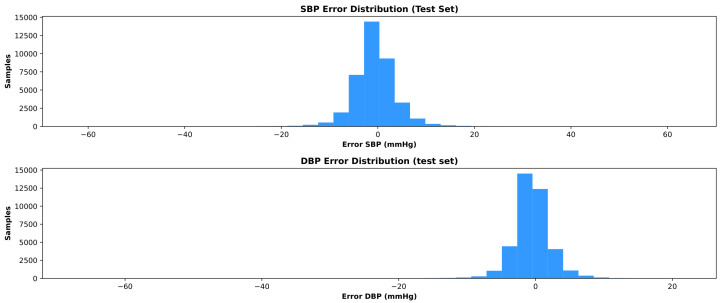
Distribution of systolic and diastolic blood pressure estimation errors obtained with the Residual 1D CNN–Slim model on the independent test set.

Figure 4 reports the distribution of true and predicted systolic and diastolic blood pressure values obtained with the Residual 1D CNN–Slim model on the test set, highlighting the close agreement between the estimated outputs and the reference measurements. This figure illustrates the distributional fidelity of the model predictions, complementing the numerical error metrics reported in Table 2. The close overlap between the true and predicted histograms indicates that the model does not systematically compress the output range, i.e., it does not suffer from the “regression to the mean” phenomenon that is commonly observed in deep-learning-based BP estimation. The slight discrepancies visible at the tails of the distributions, particularly for SBP values above 180mmHg and DBP values above 120mmHg, reflect the expected difficulty of predicting extreme blood pressure values that are underrepresented in the training data. Figure 5 shows the corresponding error distributions for the same model, which are centered around zero and exhibit limited dispersion, consistently with the quantitative results reported in Table 2.

MobileNet-1D and the Micro Temporal Convolutional Network show performance comparable to that of the baseline model, with slightly higher dispersion in systolic pressure estimation. Overall, all evaluated architectures achieve ME and SD values within commonly reported reference benchmarks on this dataset and evaluation split, while exhibiting markedly different accuracy–complexity trade-offs.

### 3.2. Quantized Inference Performance

Based on the floating-point results and architectural complexity analysis, the Baseline 1D CNN and the Residual 1D CNN–Slim were selected for quantization, as they represent complementary design points in terms of accuracy and resource requirements.

The quantization analysis was therefore conducted on these two models using full integer post-training quantization, and performance was evaluated on the same test set adopted in the floating-point analysis. The quantized models were exported in TensorFlow Lite format, which represents the effective inference representation used on resource-constrained embedded devices.

A representative dataset composed of 512 randomly selected training samples was used during calibration to estimate activation dynamic ranges. The conversion was configured to enforce full integer inference with INT8 operations and INT8 input and output tensors, without any retraining or fine-tuning after quantization.

Table 3 reports the estimation performance of the quantized models in terms of ME, SD of the signed error, and MAE for systolic and diastolic blood pressure. Model size after quantization is also reported to highlight the impact of INT8 conversion on memory footprint.

The Residual 1D CNN–Slim exhibits strong robustness to quantization, maintaining estimation accuracy and error dispersion comparable to its floating-point counterpart while achieving a substantial reduction in model size. Conversely, the Baseline CNN shows a noticeable degradation in estimation accuracy after quantization, with increased bias and variability, particularly for systolic pressure.

Specifically, the Baseline 1D CNN exhibits a substantial shift in SBP mean error from −0.27mmHg (floating-point) to +3.39mmHg (INT8), accompanied by an increase in SBP SD from 7.38 to 8.20mmHg, which exceeds the commonly adopted 8mmHg reference threshold. In contrast, the Residual 1D CNN–Slim preserves its estimation characteristics with minimal drift: the SBP ME shifts from 1.29 to 0.62mmHg, while the SBP SD changes only marginally from 4.57 to 4.68mmHg. For diastolic pressure, the pattern is consistent: the Baseline CNN shows a bias increase from 0.01 to 3.24mmHg, while the Slim model remains nearly unchanged (0.75 to 0.59mmHg). These results suggest that residual architectures, by promoting more uniform activation distributions and smoother loss landscapes, are inherently more robust to the rounding and range-compression effects induced by integer quantization. The model size reduction from 288KB (floating-point) to 166KB (INT8) further confirms the practical benefit of post-training quantization for embedded deployment.

These results indicate that architectural design plays a critical role in ensuring quantization robustness and motivate the selection of Residual 1D CNN–Slim for on-device deployment. For embedded execution, the quantized model was subsequently processed using the STM32Cube.AI toolchain, which converts the TensorFlow Lite representation into optimized C code for execution on the STM32N6 microcontroller using integer arithmetic.

### 3.3. Floating-Point vs. INT8 Consistency Analysis

To verify the numerical consistency of the quantized inference, a comparison between floating-point (FP32) and INT8 predictions was conducted on the test set. The Pearson correlation coefficient ρ was used to assess the linear agreement between the two numerical representations. The Pearson correlation coefficient is defined as follows:

(6) $$\rho =\frac{\sum_{i=1}^{N}\left(x_{i}-\bar{x}\right)\left(y_{i}-\bar{y}\right)}{\sqrt{\sum_{i=1}^{N}{\left(x_{i}-\bar{x}\right)}^{2}\cdot \sum_{i=1}^{N}{\left(y_{i}-\bar{y}\right)}^{2}}}$$

where $x_{i}$ and $y_{i}$ denote the FP32 and INT8 predictions for the *i*-th test sample, respectively, and $\bar{x}$, $\bar{y}$ are their means.

The Residual 1D CNN–Slim exhibits an excellent correspondence between FP32 and INT8 predictions, with correlation coefficients exceeding 0.99 for both systolic and diastolic blood pressure. This result confirms that the quantized model preserves the numerical behavior of the floating-point implementation, with only minor deviations attributable to reduced numerical precision.

In contrast, the Baseline 1D CNN shows a lower degree of agreement between FP32 and INT8 outputs, especially for diastolic blood pressure (ρ=0.970), accompanied by larger prediction discrepancies. These observations further highlight the superior robustness of the Residual 1D CNN–Slim architecture to aggressive quantization and support its selection for on-device deployment.

### 3.4. On-Device Evaluation on STM32N6

The STM32N6 microcontroller was selected as the target deployment platform for several reasons. First, it belongs to the latest generation of STMicroelectronics’ microcontroller family explicitly designed for edge-AI workloads, featuring an Arm Cortex-M55 core with hardware support for neural processing operations. Second, it is fully supported by the STM32Cube.AI industrial toolchain, which provides a seamless and reproducible pipeline for model conversion, optimization, and deployment from TensorFlow Lite to on-device C code. Finally, the STM32N6 offers a memory and computational profile representative of next-generation wearable and IoT health-monitoring devices, making the deployment results directly relevant to practical application scenarios.

The selected quantized model was deployed and evaluated on the target STM32N6 microcontroller to assess its on-device blood pressure estimation performance under realistic execution conditions. The model was deployed using the STM32Cube.AI toolchain and executed on the STM32N6 microcontroller operating at 300MHz. Inference was performed using the full integer (INT8) TensorFlow Lite model, and on-device predictions were compared against reference systolic and diastolic blood pressure values.

According to the STM32Cube.AI analysis report, the deployed network requires 596,853 bytes of Flash memory (including weights and runtime libraries) and 91,821 bytes of RAM, with 90,112 bytes allocated for activations.

The on-device evaluation resulted in a mean error of −0.93±4.19mmHg for systolic blood pressure and −0.65±2.91mmHg for diastolic blood pressure. The corresponding mean absolute errors were 3.20mmHg for SBP and 2.10mmHg for DBP. No violations of the physiological constraint DBP ≤ SBP were observed during on-device inference.

The average inference time measured on the microcontroller was 165.8ms per input window.

To further characterize the deployment feasibility, a sustained throughput analysis was conducted based on the measured inference time and the PPG acquisition window. Given that each input window spans 8.192s of PPG signal and the on-device inference requires 165.8ms, the inference-to-acquisition ratio is approximately 2.0%, leaving 98% of the acquisition period available for ancillary operations such as sensor data acquisition, signal preprocessing, artifact detection, wireless data transmission, or power management. This large timing margin confirms that the deployed model can operate comfortably within the real-time constraints of continuous monitoring, even under the conservative clock configuration of 300MHz, well below the maximum 800MHz supported by the STM32N6.

Regarding memory allocation, the STM32Cube.AI report indicates that the total Flash footprint of 596,853 bytes includes both the quantized network weights and the runtime inference library. The RAM requirement of 91,821 bytes is dominated by the activation buffer (90,112 bytes), which stores intermediate feature maps during layer-by-layer inference, with the remaining 1709 bytes allocated to runtime overhead. These figures confirm that the deployed model fits comfortably within the 4.2MB embedded SRAM of the STM32N6, leaving substantial memory headroom for application-level software.

These results confirm that the quantized network preserves clinically acceptable accuracy when executed on a microcontroller-class platform, achieving ME and SD values within commonly reported reference benchmarks on the adopted dataset. Moreover, the close agreement between on-device and off-device performance demonstrates that the deployment process does not introduce significant numerical degradation. Overall, the on-device evaluation validates the feasibility of performing cuffless blood pressure estimation directly on low-power embedded hardware.

## 4. Discussion and Conclusions

### 4.1. Discussion

This work investigated the feasibility of cuffless blood pressure estimation using a single photoplethysmography signal within a fully embedded, microcontroller-oriented framework. The comparative analysis of multiple convolutional neural network architectures highlighted that estimation accuracy alone is not sufficient to determine suitability for embedded deployment, as robustness to quantization and numerical stability play a critical role when operating under strict memory and computational constraints.

Consistently with previous embedded implementations, the balance between model accuracy, memory footprint, and computational cost emerges as a decisive factor for always-on wearable applications. In this regard, the results confirm that architectural compactness and residual connectivity strongly influence numerical behavior when transitioning from floating-point inference to full integer quantization.

The structured comparison presented in Section 3 can be interpreted as an ablation-like analysis that isolates the contribution of key design choices. Residual connections emerge as the single most impactful architectural feature, substantially reducing error dispersion in both floating-point and quantized regimes. Channel reduction (slimming) provides a highly favorable trade-off, preserving most of the accuracy of the full residual model at a fraction of the parameter count. Depthwise separable convolutions, while extremely effective for model compression, appear to compromise regression robustness in this task. These observations suggest that architecture selection for edge deployment should prioritize quantization-aware design over raw floating-point accuracy or minimal model size.

Among the evaluated models, the Residual 1D CNN–Slim architecture provided the most favorable trade-off between estimation accuracy, model complexity, and robustness to reduced numerical precision. While larger residual networks achieve slightly improved floating-point performance, their memory footprint limits practical deployment on microcontroller-class devices. Conversely, simpler baseline architectures exhibit increased sensitivity to INT8 quantization, leading to bias amplification and accuracy degradation.

The quantized and on-device evaluations demonstrate that a compact convolutional neural network can preserve ME and SD values within commonly reported reference benchmarks even under aggressive numerical constraints, confirming that reliable cuffless blood pressure estimation can be achieved without multimodal sensing or cloud-based processing. The on-device results obtained on the STM32N6 microcontroller show that the complete inference pipeline can be executed directly on a microcontroller-class platform, remaining well within the acquisition window of the physiological signal.

It should be emphasized that the favorable error metrics reported in this study reflect window-level performance on a retrospective dataset with random partitioning, and should not be interpreted as evidence of clinical readiness. The reported ME and SD values represent a necessary but not sufficient condition for clinical applicability: prospective, subject-level validation on diverse populations using certified reference devices would be required before any clinical deployment of the proposed system. The primary contribution of this work is methodological—demonstrating that quantization robustness is an essential design criterion and that on-device inference is feasible—rather than clinical.

As previously highlighted in embedded BP estimation studies, the availability of a large timing margin facilitates the integration of additional operations, such as sensor handling, artifact control, or power-management strategies, without compromising real-time performance. Although the measured inference latency reflects the constraints of the current deployment configuration, it confirms the feasibility of continuous on-device inference in practical wearable scenarios.

Several convolutional neural network approaches for cuffless blood pressure estimation have been reported in the literature [16], frequently evaluated on desktop-class processors or general-purpose embedded platforms. However, differences in dataset partitioning strategies, preprocessing pipelines, quantization schemes, and hardware configurations limit direct quantitative comparison across studies. The present work therefore emphasizes architecture-dependent quantization robustness and validated deployment on a microcontroller-class device under strict memory and computational constraints, rather than claiming direct performance superiority over previously reported systems.

To contextualize the present work within the existing literature, Table 4 summarizes the key characteristics of representative prior studies addressing PPG-based blood pressure estimation with an edge or embedded focus. The comparison is organized along five dimensions: input modality, neural architecture, target deployment platform, quantization strategy, and reported estimation accuracy.

Among the listed studies, Sun et al. [16] is the most closely related to the present work, as it investigates the deployment of multiple CNN architectures on microcontroller-class devices using pruning and quantization. However, their evaluation relies on general-purpose edge platforms (Arduino, ESP32, Raspberry Pi) without reporting quantization-induced accuracy degradation or performing a systematic comparison between floating-point and integer models. Bernard et al. [17] proposed a hybrid CNN–LSTM architecture achieving sub-2mmHg errors for SBP, but the model was deployed on an IoT-oriented computing device rather than a resource-constrained MCU, and no quantization analysis was reported. Ali et al. [19] deployed a convolutional autoencoder on an Arduino Nano 33 BLE Sense and reported real-time MCU-based performance; however, the model operates on extracted PPG features rather than raw waveforms, and no quantization robustness analysis was provided. Joseph and T.S. [4] presented a hardware-oriented LSTM-based approach for wearable BP prediction, but required both ECG and PPG inputs.

The present work differs from these studies in three key respects: (i) it provides a systematic, architecture-level comparison of quantization robustness, demonstrating that floating-point accuracy is a poor predictor of post-quantization performance; (ii) it validates the complete pipeline on a microcontroller-class platform (STM32N6) using an industrial deployment toolchain; and (iii) it relies exclusively on single-channel raw PPG input without feature engineering or multimodal sensing. These aspects collectively distinguish the proposed framework from prior work and highlight the importance of hardware-aware model selection for embedded physiological monitoring.

#### Limitations

Several limitations of this study should be acknowledged. First, the evaluation is retrospective in nature: all experiments were conducted on a publicly available dataset (UCI Cuff-Less Blood Pressure Estimation), and no prospective clinical validation was performed. Second, the dataset is limited in size and demographic diversity, comprising 500 valid recordings without detailed information on subject age, sex, ethnicity, or comorbidities, which limits the generalizability of the results to broader populations. Third, as discussed in Section 2, the adopted data partitioning strategy is random rather than subject-wise, which may lead to optimistic error estimates due to data leakage from overlapping windows originating from the same subject. Fourth, each architecture was trained using a single fixed random seed; while this ensures reproducibility, it does not capture the variability across different initializations that multi-seed evaluation would provide. Fifth, no external validation on independent datasets was performed, which would be necessary to assess the robustness and transferability of the models beyond the training distribution. Sixth, direct power consumption measurements during on-device inference were not conducted in this study; energy estimates would require dedicated hardware instrumentation for precise characterization. Seventh, the same held-out test set was used both for the floating-point comparison among architectures and for the subsequent evaluation of the quantized and deployed models; although the selection of the two quantization candidates was driven by a joint accuracy–complexity criterion rather than by raw test accuracy, a mild form of model-selection bias cannot be excluded, and a fully independent test set would be required for rigorous clinical performance benchmarking. Each of these limitations represents an important direction for future work toward clinical translation.

### 4.2. Conclusions

This paper presented an end-to-end approach for cuffless blood pressure estimation based on a single photoplethysmography signal, explicitly targeting deployment on resource-constrained microcontroller platforms. A systematic evaluation of multiple neural network architectures was conducted to identify models capable of balancing estimation accuracy, computational efficiency, and robustness to quantization.

The experimental results demonstrate that a compact residual convolutional network can maintain clinically acceptable performance after full integer quantization and during on-device execution, without relying on multimodal inputs or cloud-based processing. The on-device validation on the STM32N6 microcontroller confirms that accurate blood pressure estimation can be achieved entirely at the edge, achieving ME and SD values within commonly reported reference benchmarks while operating under strict memory and latency constraints.

Overall, this work bridges the gap between algorithmic development and real-time embedded deployment, showing that careful hardware–software co-design enables reliable physiological monitoring in always-on wearable systems. Future work will focus on further optimizing inference latency, integrating on-device preprocessing, and extending validation to prospective clinical datasets across diverse populations.

Beyond blood pressure estimation, the proposed framework is directly applicable to a broader range of wearable health-monitoring scenarios, including continuous cardiovascular risk stratification in smartwatches, point-of-care screening in primary care settings, and remote patient monitoring in telemedicine. The edge-AI approach is also generalizable to other PPG-derived physiological parameters, such as heart rate variability, peripheral oxygen saturation (SpO_2_), and respiratory rate estimation, as well as to other biosignal processing tasks (e.g., EMG, EDA) on microcontroller-class hardware. The demonstrated feasibility of deploying compact quantized neural networks on low-power MCUs supports the vision of fully autonomous, privacy-preserving wearable systems capable of multi-parameter health monitoring without cloud dependency.

## Figures and Tables

**Table 1:** Architectural and training characteristics of the evaluated neural network models.

Architecture	Input Shape	Main Architectural Features	Loss	Optimizer	LR	Weight Decay	Batch Size	Training Strategy
Baseline 1D CNN	(1024, 1)	Stacked Conv1D + dense regression head	MSE	Adam	$1\times 10^{-3}$	–	128	Fixed epochs (400)
Residual 1D CNN	(1024, 1)	Residual blocks with increasing channels	Huber	AdamW	$8\times 10^{-4}$	$1\times 10^{-4}$	128	Early stopping
Residual 1D CNN–Slim	(1024, 1)	Compact residual architecture with GAP	Huber	AdamW	$8\times 10^{-4}$	$1\times 10^{-4}$	128	Early stopping
MobileNet-1D	(1024, 1)	Depthwise separable 1D convolutions	Huber	AdamW	$8\times 10^{-4}$	$1\times 10^{-4}$	128	Early stopping
Micro Temporal Convolutional Network	(1024, 1)	Dilated temporal convolutions in residual structure	Huber	AdamW	$8\times 10^{-4}$	$1\times 10^{-4}$	128	Early stopping

**Table 2:** Floating-point blood pressure estimation performance.

Architecture	SBP ME ± SD (mmHg)	SBP MAE (mmHg)	DBP ME ± SD (mmHg)	DBP MAE (mmHg)	# Params	Model Size (KB)
Baseline 1D CNN	−0.27±7.38	5.13	0.01±4.68	3.17	33,826	137
Residual 1D CNN	1.55±3.35	2.59	0.36±2.24	1.45	554,498	1094
Residual 1D CNN–Slim	1.29±4.57	3.30	0.75±3.25	2.10	140,034	288
MobileNet-1D	0.12±7.94	5.73	0.44±4.39	3.04	10,738	37
Micro TCN	2.18±7.05	5.42	0.20±4.09	2.67	41,698	105

**Table 3:** TFLite INT8 blood pressure estimation performance on the independent test set.

Architecture	SBP ME ± SD (mmHg)	SBP MAE (mmHg)	DBP ME ± SD (mmHg)	DBP MAE (mmHg)	Model Size (KB)
Baseline 1D CNN	3.39±8.20	6.47	3.24±4.99	4.28	43
Residual 1D CNN–Slim	0.62±4.68	3.22	0.59±3.26	2.07	166

**Table 4:** Comparison with representative prior PPG-based BP estimation studies with edge or embedded focus.

Study	Input	Architecture	Target Platform	Quantization	SBP MAE (mmHg)	DBP MAE (mmHg)
Sun et al. [16]	PPG	AlexNet, ResNet, MobileNet, etc.	Arduino, ESP32, Raspberry Pi	Pruning + Quant.	AAMI compliant ^*a*^	
Bernard et al. [17]	PPG	CNN–LSTM (hybrid)	Medical Edge (IoT)	None reported	<2	<2
Ali et al. [19]	PPG features	Conv. autoencoder	Arduino Nano 33 BLE Sense	TFLite (TinyML)	2.25 ^*b*^	5.01 ^*b*^
Joseph & T.S. [4]	ECG + PPG	LSTM DNN	Wearable (HW)	Split matrix	—	—
Botrugno et al. [21]	Multi-λ PPG	CNN–SVR	Desktop	None reported	—	—
This work	PPG	Residual 1D CNN–Slim	STM32N6 MCU	INT8 PTQ	3.20 ^*c*^	2.10 ^*c*^

^*a*^ Per-architecture MAE not individually reported; overall AAMI/BHS compliance stated. ^*b*^ MCU-based real-time results on 8 volunteers. ^*c*^ On-device (STM32N6) results on the independent test set.

## Data Availability

The datasets generated and analyzed during the current study are available from the corresponding author upon reasonable request.
